# ProbC: joint modeling of epigenome and transcriptome effects in 3D genome

**DOI:** 10.1186/s12864-022-08498-5

**Published:** 2022-04-09

**Authors:** Emre Sefer

**Affiliations:** Department of Computer Science, Ozyegin University, Istanbul, Turkey

**Keywords:** Epigenetics, Hi-C, Micro-C, Machine learning, Chromatin organization

## Abstract

**Background:**

Hi-C and its high nucleosome resolution variant Micro-C provide a window into the spatial packing of a genome in 3D within the cell. Even though both techniques do not directly depend on the binding of specific antibodies, previous work has revealed enriched interactions and domain structures around multiple chromatin marks; epigenetic modifications and transcription factor binding sites. However, the joint impact of chromatin marks in Hi-C and Micro-C interactions have not been globally characterized, which limits our understanding of 3D genome characteristics. An emerging question is whether it is possible to deduce 3D genome characteristics and interactions by integrative analysis of multiple chromatin marks and associate interactions to functionality of the interacting loci.

**Result:**

We come up with a probabilistic method ProbC to decompose Hi-C and Micro-C interactions by known chromatin marks. ProbC is based on convex likelihood optimization, which can directly take into account both interaction existence and nonexistence. Through ProbC, we discover histone modifications (H3K27ac, H3K9me3, H3K4me3, H3K4me1) and CTCF as particularly predictive of Hi-C and Micro-C contacts across cell types and species. Moreover, histone modifications are more effective than transcription factor binding sites in explaining the genome’s 3D shape through these interactions. ProbC can successfully predict Hi-C and Micro-C interactions in given species, while it is trained on different cell types or species. For instance, it can predict missing nucleosome resolution Micro-C interactions in human ES cells trained on mouse ES cells only from these 5 chromatin marks with above 0.75 AUC. Additionally, ProbC outperforms the existing methods in predicting interactions across almost all chromosomes.

**Conclusion:**

Via our proposed method, we optimally decompose Hi-C interactions in terms of these chromatin marks at genome and chromosome levels. We find a subset of histone modifications and transcription factor binding sites to be predictive of both Hi-C and Micro-C interactions and TADs across human, mouse, and different cell types. Through learned models, we can predict interactions on species just from chromatin marks for which Hi-C data may be limited.

## Introduction

The accumulating evidence suggests that 3D nuclear architecture is important for the gene expression regulation, key element in transcriptional regulation [1], and it is firmly connected to genome’s function. The 3D chromatin structure brings DNA regions separated by great genomic distance into spatially closer sections, organizing interactions between regulatory elements and genes. As an example, enhancer and corresponding transcription factors are brought within close distance of beta-globin locus genes and impact their expression by folding. Similarly, the disruption of an eQTL in FTO gene turns on a pro-obesity phenotype and increases the expression of distant genes IRX3 and IRX5 in preadipocytes [2]. Thus, modeling and computing genome’s 3D shape is crucial to completely understand the functioning of cells.

Multiple chromosome conformation capture experimental techniques, for instance Hi-C, have remarkably improved our comprehension of 3D chromatin structure [3]. Hi-C methods generate chromatin inter- actions throughout the genome and contact frequencies between pair of loci. The number of chromatin interactions between pair of loci is a measure of how such loci pair are near each other in 3D space. At a given resolution, Hi-C experiment returns interaction frequency matrix which represent the cross-linking prevalence among DNA restriction fragments as output. Hi-C experiments have revealed A and B compartments which correspond to the partition of spatial genome into open and closed chromatin [4]. Among these compartments, A compartment is related to easily accessible, transcriptionally active euchromatin. Similarly, B compartment is related to condense, transcriptionally inactive heterochromatin. Resulting analysis of the Hi-C interaction matrix at a higher resolution has discovered topologically-associated domains (TADs) which are frequently-interacting, sequential, closely-located interaction matrix areas [5]. TADs are pervasive genome organization unit which are stable attributes of Hi-C matrices. TADs match greatly with cellular differentiation and long-range transcriptional control [6].

Similarly, Micro-C is a Hi-C based method where micrococcal nuclease is used instead of restriction enzymes to fragment chromatin, enabling nucleosome resolution chromosome folding maps. Chromatin is fragmented to mononucleosomes, thereby increasing both fragment density as well as uniformity of spacing. Crucially, Micro-C overcomes the current resolution gap of Hi-C at the fine scale, which now allows us to investigate more detailed chromatin structures. Hi-C and Micro-C technologies have led to the intuitive observation of the genome structure of human and mouse [7, 8], and yeast [9]. During this text, we will use the term Hi-C to refer both Hi-C and its very high nucleosome resolution varint Micro-C experiments.

Epigenetics is quite important in understanding the basic cellular pathways and processes occurring in chromatin (DNA repair, splicing, transcription, and replication). Even though Hi-C does not directly depend on the binding of specific antibodies, previous analysis has revealed enriched interactions and domain structures around multiple chromatin marks; histone modifications and transcription factor binding sites (TFBSs) [5, 10]. Interactions between these 1D chromatin marks are important in three-dimensional genome structure even if these interactions causal direction is unknown. For instance, histone modification H3K27me3 is significantly diminished within TAD boundaries [11] whereas insulator proteins, modifications H3K27ac and H3K4me3 are enriched inside TAD boundaries. TAD boundaries are stabilized by the cohesin complex and are generally enriched in architectural proteins such as transcriptional repressor CCCTC-binding factor (CTCF) [5, 12]. Moreover, CTCF and cohesin play an important role in chromatin loop formation [13] and in establishment of FIREs [14].

Despite such analyses, the ability of histone modifications affect 3D genome shape through the binding sites remains poorly understood across species, cell types, and cell cycles. This can be partially due to the fact that the previous research on the relationships between histone modifications and genomic structures have frequently considered each modification independently, ignoring their joint quantitative effects. However, it has been previously shown that multiple modifications are jointly important in genome shape. For instance, both H3K4me3 and H3K27me3 are known to be effective in TAD formation [5, 15] which fail to explain TADs when considered independently. Additionally, we do not fully know the degree of importance of the relationships between the histone modifications across species and cell types. Similarly, we also do not know whether a subset of histone modifications are primarily important in explaining observed Hi-C interactions, and thus 3D genome shape.

In this paper, we focus on the problem of identifying the relationships between high-order chromatin interactions and chromatin marks; epigenetic and transcription-related marks. Distinctively, we aim to understand and predict how Hi-C and Micro-C interactions are formed as a result of these marks and interactions within them. We propose a generative probabilistic method PROBC to decompose genome-wide Hi-C and Micro-C interactions by chromatin marks. PROBC estimates interaction probabilities between chromatin marks based on convex likelihood optimization, which can directly take into account both the interaction existence and nonexistence. Through PROBC, we systematically find 4 histone modifications (H3K27ac, H3K9me3, H3K4me3, H3K4me1) and CTCF to be highly predictive of most Hi-C and Micro-C interactions across cell types and mammals when considered together. Similarly, we also identify subset of transcription factor binding sites to be highly predictive of Hi-C and Micro-C interactions. We complete the missing Hi-C and Micro-C interactions and predict intra-chromosomal interactions at a very high resolution. Identified sparse set of chromatin marks account for a large proportion of the accuracy of Hi-C prediction, matching with their known roles, which fail to predict Hi-C and Micro-C interaction when considered independently. These marks and interaction probabilities between them inferred by PROBC can also be interpreted as latent biases in Hi-C and Micro-C experiments. We also discover that chromatin marks are conserved across cell types and species in a robust way: PROBC trained on embryonic stem cells work on IMR90 cells quite accurately, and PROBC trained on mouse keep working well on human. However, we also interpret our cross-species and cross-cell types results carefully as suggested in [16].

In the rest of the paper, we start by formally defining PROBC problem while presenting an optimal method to understand the relationships between chromatin marks and genome shape through Hi-C and Micro-C. Then, we infer a subset of chromatin marks effective in explaining Hi-C and Micro-C interactions. Lastly, we present results on the prediction of Hi-C and Micro-C interactions and TADs on the same species as well as across species and cell types. Overall, our contributions can be summarized as follows: 1- We come up with a novel formulation for the problem of systematically identifying mark interactions and biases in Hi-C and Micro-C data throughout the genome at a very high resolution, 2- We propose a rigorous method PROBC with optimality guarantee which is also the first method to identify the importance of chromatin marks in higher resolution Micro-C dataset, 3- We show that most of the inferred epigenetic and transcription-related marks and relationships between them exist consistently across different mammals and cell types regardless of the experiment type, 4- We exploit such relationships to predict genome organization of mammals without the interaction data, through epigenetic and transcription-related marks. We find PROBC to outperform the state-of-the-art methods in Hi-C and Micro-C interaction prediction across most chromosomes. As a result, we find that identified chromatin marks and interactions between them carry sufficient information to predict genome structures. Additionally, all predictions made by PROBC give an efficient guide to explore the organization of chromatin.

### Related work

Previous research has analyzed various genomic structures through epigenetic marks without taking the interactions between the marks into account. Developed methods analyze the data without defining a generative model. Rao et al. [3] and Ernst and Kellis [17] analyzed how enhancers, CTCF, histone modifications are distributed across the genome in terms of Hi-C chromosomal contacts. On the other hand, Al Bkhetan and Plewczynski [18] uses a statistical learning framework to predict 3D chromatin looping interactions inside TADs from transcription factor profiles and epigenomics. Similarly, Ashoor et al. [19] predicts genomic sub-compartments from Hi-C chromatin interaction data by unsupervised graph embedding. Lastly, Sefer and Kingsford [15] and Libbrecht et al. [20] has analyzed the histone modifications impact in TAD prediction without focusing on Hi-C interaction prediction.

In another set of research, deep non-generative models have been used to analyze epigenetic modifications on Hi-C. Di Pierro et al. [21] predicts subcompartment annotations from plethora of epigenetic modifications and protein binding sites by using a deep neural network only in GM12878 cells. However, the application of their deep learning approach to most other cell types is limited as most cell types do not have as many ChIP-seq datasets as GM12878 does. Among similar algorithms, SNIPER [22] reveals Hi-C subcompartments by imputinvg inter-chromosomal chromatin interactions via autoencoders. Li et al. [23] predicts interactions only between regulatory elements by using a bootstrapping deep learning model. Their method integrates chromatin accessibility and genome sequences data without taking epigenetic modifications into account. Similarly, Trieu et al. [24] predicts the impact of solely non-coding sequence variants on three-dimensional chromatin shape by a deep learning approach. On the other hand, RIPPLE [25] predicts enhancer-promoter interactions in a cell line-specific manner by combining Hi-C with regulatory genomics datasets.

The most similar work to ours is HiC-Reg, which predicts Hi-C interactions from one-dimensional regulatory signals by using a random forest based regression model [26]. HiC-Reg can capture nonlinear interactions in its random forest model, and it performs accurately in most datasets. However, HiC-Reg is not a generative model. Even though random forest models do a good job at classification, they do not perform as well for regression problem since they don’t estimate past the range of the training dataset accurately together with them possibly overfitting noisy datasets. Additionally, HiC-Reg is not regularized. Among similar existing work, EpiTensor [27] and Rambutan [28] construct 3D genome shape from one dimensional chromatin signals. EpiTensor is capable of extracting meaningful co-variation patterns from histone modifications, chromatin accessibility and RNA-seq, but its decomposition requires a single run jointly across different cell types. Rambutan proposes a deep convolutional neural network that predicts Hi-C contacts at a very high resolution using nucleotide sequence and DNase I signal as inputs. Another recent work [29] develops an adversarial training-based approach to predict Hi-C interaction maps from 1D epigenomic signals. We found PROBC to perform better than HiC-Reg, and Rambutan for most of the tested chromosomes.

Our work is different than the existing work in terms of the following points: 1- A number of the existing research such as [3] treat each histone modification independently without considering the cellular interactions and dynamics between these modifications, 2- The existing methods don’t come up with generative models of Hi-C interactions by epigenetic and transcription-related marks, so interpretability of the relations between these marks and interactions in these methods is limited, 3- Some of the existing methods are not provably optimal, and they do not quantify the degree of relationships between mark pairs in explaining Hi-C interactions, and 4- The existing methods do not identify the importance of chromatin marks in high resolution Micro-C dataset, but focus only on lower resolution Hi-C dataset. Therefore, they can not identify the most explanatory set of epigenetic and transcription-related elements.

## Methods

### Problem definition

As discussed in [30], Hi-C provides us set of interactions between restriction sites on overall genome. More formally, let *R* be the set of restriction sites over considered genome, Hi-C provides us an undirected interaction graph *G*=(*V*=*R*,*E*) where *E*={*E*_*uv*_, *u*<*v*∈*R*^2^} is set of interactions between restriction sites and *E*_*uv*_ is the number of interactions between *u* and *v*. We can analyze these interactions in following 2 ways: 1- We can focus on working at restriction site level where *G* is an unweighted graph and every node is a restriction site, or 2- We bin the interactions at a given resolution and examine the resulting graph $\phantom {\dot {i}\!}G^{'}=(V^{'}=R^{'}, E^{'})$ where $\phantom {\dot {i}\!}R^{'}$ defines fixed-length genomic intervals (called a bin) without an overlap, and every edge $E^{'}_{uv}$ represents the total interaction count between restriction sites of bins *u* and *v*.

Let *M* be set of genomic marks such as histone modifications, transcription factor binding sites that are shown to be linked to several Hi-C interaction structures [5]. These marks are candidates to explain observed Hi-C interactions and associated biases. We define $c^{v}_{m}$ to be the number of mark *m*∈*M* around restriction site *v* that could take binary values if the data is not binned; mark *m* either exists or not around *v*. Let $N[v] = \{(m, c^{v}_{m}),\,|\,m \in M, c^{v}_{m} > 0\}$ be set of mark counts around restriction site *v*. After binning, $\phantom {\dot {i}\!}N[v^{'}] = \{(m, \sum _{k=1}^{t}\,c^{k}_{m}),\,|\,m \in M, \sum _{k=1}^{t}\,c^{k}_{m} > 0\}$ where bin $\phantom {\dot {i}\!}v^{'} = \{v_{1}, v_{2}, \ldots, v_{t}\} \in R^{'}$ includes *t* restriction sites. Given *N*={*N*[*v*], *v*∈*R*} (or $\phantom {\dot {i}\!}N = \{N[v^{'}],\,v^{'} \in R^{'}\}$ if the data is binned), we present the problem 1 to infer set of chromatin marks by which Hi-C data can be understood:

#### **Problem 1**

PROBC: Given Hi-C interaction graph *G* and mark data *N* over a genome as input, we estimate the interaction probabilities between all mark pairs that jointly explain *G*.

Problem is defined similarly when the input Hi-C interaction data and mark data are binned at a provided resolution. PROBC identifies the most likely subset of mark interactions jointly explaining both interacting and non-interacting genomic regions. By inferring the subset of mark interactions that are important rather than just marks, PROBC can provide more insight into the relation between interactions between marks and the formation of genome shape. PROBC can also take into account the prior information about marks such as sparsity, and/or block structure of the effective marks. Identified subset of marks and interaction probabilities between marks can also be interpreted as possible explanations of Hi-C biases.

### PROBC: generative probabilistic model to explain hi-C interactions by mark pairs

We come up with a generative penalized maximum likelihood based formulation to model the mark effects in Hi-C considering both interactions and non-interactions. Let *x*_*mn*_ be the probability of interaction between marks *m* and *n*, and **X**={*x*_*mn*_, (*m*,*n*)∈*M*^2^} be the symmetric matrix of interaction probabilities between mark pairs. We assume these interaction probabilities between mark pairs **X** to be same across all restriction sites which is a global characteristic of genome embedding rather than being a local feature. This assumption is discussed in previous studies [3, 5, 20, 31, 32]. Dixon et al. [5], Huang et al. [31] and Fortin and Hansen [32] find distinct patterns of histone marks to be same around topologically associated domain (TAD) boundaries, and A/B compartments throughout the genome. Rao et al. [3] and Libbrecht et al. [20] discuss the similarity of marks across TAD boundaries in different species and cell types.

Given interaction data between restriction sites *G*, its likelihood is:

1 $$ \begin{aligned} \mathcal{L}(\mathbf{X}, G) = P(G | \mathbf{X}) &= \prod_{(u, v) \in E} P(E_{uv} | \mathbf{X}) \\ &\quad\prod_{(u, v) \notin E} \left(1 - P\left(E_{uv} | \mathbf{X} \right) \right) \end{aligned}  $$

due to independence of Hi-C interactions between restriction sites where *P*(*E*_*uv*_|**X**) is the probability of observing an interaction between restriction sites *u* and *v* given mark interaction probabilities **X**. As you notice, Eq. 1 is a multiplication over both interacting and non-interacting restriction site pairs. We can express *P*(*E*_*uv*_|**X**) as in:

2 $$ {}P(E_{uv} | \mathbf{X}) = 1- \prod_{(m, c_{m}) \in N[u]}\,\prod_{(n, c_{n}) \in N[v]}\left(1-x_{mn}\right)^{c_{m}c_{n}}  $$

Note that this simple process already ensures that pairs of restriction sites that have relevant mark pairs are more likely to interact in Hi-C. This is due to the fact that restriction sites that share multiple mark pairs receive multiple chances to create Hi-C interaction.

Although minimizing negative logarithm of the likelihood (1) is non-convex, we can transform it into a convex problem by changing the variables $\phantom {\dot {i}\!}x_{uv} = 1- e^{-z_{uv}}$. We also add sparsity and low-rank regularization terms. The sparsity of mark interaction probabilities implies that only a small fraction of mark pairs affect Hi-C interactions. Furthermore, we want to ensure block diagonal/communities structure of mark interactions matrix which implies low-rank structures. Block diagonal structure implies that the probability of interaction between marks *m* and *n* (*x*_*mn*_) to be high when *x*_*mk*_ and *x*_*nk*_ are also high. Overall, we optimize the following objective by imposing both sparse and low-rank regularization on transformed variables **Z**={*z*_*mn*_, (*m*,*n*)∈*M*^2^}:

3 $$ \operatornamewithlimits{argmin}_{\mathbf{Z} \ge 0} -\log(\mathcal{L}(\mathbf{Z}, G))\,+\,\lambda_{1} \|\mathbf{Z}\|_{*} \,+\,\lambda_{2} \|\mathbf{Z}\|_{1}  $$

where $\log (\mathcal {L}(\mathbf {Z}, G))$ can be expressed as:

4 $$ {}\begin{aligned} \log(\mathcal{L}(\mathbf{Z}, G) &= \sum_{(u, v) \in E}\,\log\left(1- \exp^{-C_{u}^{T}\mathbf{Z}C_{v}}\right)\\ &\quad- \sum_{(u, v) \notin E} C_{u}^{T}\mathbf{Z}C_{v} \end{aligned}  $$

where $C_{u} = [c^{u}_{m},\,m \in M]$ is a *M*×1 binary vector of mark counts/existence at site *u*. $\|\mathbf {Z}\|_{*} = \sum _{i=1}^{rank \mathbf {Z}} \sigma _{i}$ is matrix **Z**’s nuclear norm, that is sum of **Z**’s singular values. Matrix’s nuclear norm has been applied for efficient estimation of low-rank matrices [33]. Additionally, $\|\mathbf {Z}\|_{1} = \sum _{u, v} |z_{uv}|$ is matrix **Z**’s *l*_1_, that is used to impose the sparsity of **Z**. This means we can ignore the effect of mark pairs in Hi-C which are 0. The parameters *λ*_1_ and *λ*_2_ control the strength of the regularization terms. The probability constraints 0≤*x*_*uv*_≤1 turn into *z*_*uv*_≥0. Problem 3 is convex as proven in Theorem 1. This means we can find globally optimal solution by using efficient algorithms as described in next section.

#### **Theorem 1**

Objective 3 is convex.

#### *Proof*

Linear norm and nuclear norm is convex. Concavity of Eq. 4 depends on concavity of the additive expressions. $\sum _{(u, v) \notin E} C_{u}^{T}\mathbf {Z}C_{v}$ is concave as it is linear expression of *Z*. Let $C_{\prime } = C_{u}C_{v}^{T}$, $\log \left (1- \exp ^{-C_{\prime }\circ \mathbf {Z}}\right)$ is concave as its hessian matrix $\frac {-C_{\prime }^{T}C_{\prime }}{\left (1- \exp ^{-C_{u}^{T}\mathbf {Z}C_{v}}\right)^{2}}$ is negative semidefinite. As Eq. 4 is concave, its negation in Eq. 3 is convex. □

#### Efficient optimization

Objective function in Eq. 3 is generally difficult to optimize since it is non-differentiable. We use Alternating Direction Method of Multipliers (ADMM) idea [34] to solve the problem, which has reasonably good convergence properties derived from more general Douglas-Rachford splitting approach. The optimization problem above is converted to multiple subproblems which are easier to solve by ADMM framework. Specially, we first present 2 auxiliary variables *Z*_1_, *Z*_2_ and convert the optimization problem in Eq. 3 to an equivalent form:

5 $$ {}\begin{aligned} & \min_{\mathbf{Z} \ge 0, \mathbf{Z_{1}}, \mathbf{Z_{2}}}\, -\log(\mathcal{L}(\mathbf{Z}, G))\,+\,\lambda_{1} \|\mathbf{Z_{1}}\|_{*} \,+\,\lambda_{2} \|\mathbf{Z_{2}}\|_{1} \\ & \hspace{0.5cm} s.t. \hspace{1cm} \mathbf{Z} = \mathbf{Z_{1}}, \, \mathbf{Z} = \mathbf{Z_{2}} \end{aligned}  $$

In Alternating Direction Method of Multipliers, we optimize the augmented Lagrangian of the problem above which can be written as:

6 $$ {}\begin{aligned} \mathcal{L}_{\rho} &= -\log(\mathcal{L}(\mathbf{Z}, G))\,+\,\lambda_{1} \|\mathbf{Z_{1}}\|_{*} \,+\,\lambda_{2} \|\mathbf{Z_{2}}\|_{1}\\ &\quad+\!\rho\, \text{trace}\left(\mathbf{U_{1}}^{T}(\mathbf{Z}-\mathbf{Z_{1}})\right) \,\,+\,\, \rho\,\text{trace}\left(\mathbf{U_{2}}^{T}(\mathbf{Z}-\mathbf{Z_{2}})\right)\\ &\quad+\,\frac{\rho}{2}\left(\left\lVert\mathbf{Z} - \mathbf{Z_{1}}\right\rVert^{2} + \left\lVert\mathbf{Z} - \mathbf{Z_{2}}\right\rVert^{2} \right) \end{aligned}  $$

where ‖.‖ denotes the Frobenius norm and *ρ*>0 is the penalty parameter. The matrices *U*_1_ and *U*_2_ are the dual variables affiliated with the constraints **Z**=*Z*_1_ and **Z**=*Z*_2_, respectively. We can solve augmented Lagrangian in Eq. 6 by following the iterative steps below:

7 $$\begin{array}{*{20}l} & \mathbf{Z}^{k+1} = \operatornamewithlimits{argmin}_{\mathbf{Z} \ge 0}\, \mathcal{L}_{\rho}\left(\mathbf{Z}^{k}, \mathbf{Z_{1}}^{k}, \mathbf{Z_{2}}^{k}, \mathbf{U_{1}}^{k}, \mathbf{U_{2}}^{k}\right)  \end{array} $$

8 $$\begin{array}{*{20}l} & \mathbf{Z_{1}}^{k+1} = \operatornamewithlimits{argmin}_{\mathbf{Z_{1}}}\, \mathcal{L}_{\rho}\left(\mathbf{Z}^{k+1}, \mathbf{Z_{1}}^{k}, \mathbf{Z_{2}}^{k}, \mathbf{U_{1}}^{k}, \mathbf{U_{2}}^{k}\right)  \end{array} $$

9 $$\begin{array}{*{20}l} & \mathbf{Z_{2}}^{k+1} = \operatornamewithlimits{argmin}_{\mathbf{Z_{2}}}\, \mathcal{L}_{\rho}\left(\mathbf{Z}^{k+1}, \mathbf{Z_{1}}^{k}, \mathbf{Z_{2}}^{k}, \mathbf{U_{1}}^{k}, \mathbf{U_{2}}^{k}\right)  \end{array} $$

10 $$\begin{array}{*{20}l} & \mathbf{U_{1}}^{k+1} = \mathbf{U_{1}}^{k} \,+\,\left(\mathbf{Z}^{k+1} \,-\, \mathbf{Z_{1}}^{k+1}\right)  \end{array} $$

11 $$\begin{array}{*{20}l} & \mathbf{U_{2}}^{k+1} = \mathbf{U_{2}}^{k} \,+\,\left(\mathbf{Z}^{k+1} \,-\, \mathbf{Z_{2}}^{k+1}\right)  \end{array} $$

This sequential scheme let us to separate these variables and optimize them independently one by one. Next, we describe the optimization problem for *Z*_1_ and *Z*_2_. Then, we discuss the algorithm that optimizes **Z**.

#### Solving for *Z*_1_ and *Z*_2_:

When solving for *Z*_1_ in Eq. 8, the relevant $\mathcal {L}_{\rho }$ terms are $\lambda _{1} \|\mathbf {Z_{1}}\|_{*} \,+\,\rho \text {trace}\left (\left (\mathbf {U_{1}}^{k}\right)^{T}\left (\mathbf {Z}^{k+1}-\mathbf {Z_{1}}\right)\right)\,+\,\frac {p}{2}\left (\left \lVert \mathbf {Z}^{k+1} - \mathbf {Z_{1}}\right \rVert ^{2}\right.$ which becomes:

12 $$ {}\mathbf{Z_{1}}^{k+1} \,=\, \operatornamewithlimits{argmin}_{\mathbf{Z_{1}}}\,\lambda_{1} \|\mathbf{Z_{1}}\|_{*} \,+\,\frac{p}{2}\left\lVert\mathbf{Z}^{k+1} - \mathbf{Z_{1}} + \mathbf{U_{1}}^{k}\right\rVert^{2}  $$

This problem has a closed form solution:

13 $$ \mathbf{Z_{1}}^{k+1} = S_{\frac{\lambda_{1}}{p}}\,\left(\mathbf{Z}^{k+1}\,+\,\mathbf{U_{1}}^{k}\right)  $$

where soft-thresholding function *S*_*α*_(**Q**) is *S*_*α*_(**Q**)=**U**diag((*σ*_*i*_−*α*)_+_)**V**^*T*^ for matrix **Q** with corresponding Singular Value Decomposition **Q**=**U**diag(*σ*_*i*_)**V**^*T*^. Similarly, the optimization for *Z*_2_ can be expressed as follows:

14 $$ {}\mathbf{Z_{2}}^{k+1} \,=\, \operatornamewithlimits{argmin}_{\mathbf{Z_{2}}}\,\lambda_{2} \|\mathbf{Z_{2}}\|_{1} \,+\,\frac{p}{2}\left\lVert\mathbf{Z}^{k+1} - \mathbf{Z_{2}} + \mathbf{U_{2}}^{k}\right\rVert^{2}  $$

which is a closed form solution. The corresponding (*Z*_2_^*k*+1^)_*ij*_ entry is defined as below according to **Z**^*k*+1^+*U*_2_^*k*^ where *sgn* is sign function:

15 $$ (\mathbf{Z_{2}}^{k+1})_{ij} = \begin{cases} 0 & \left|\left(\mathbf{Z}^{k+1} + \mathbf{U_{2}}^{k}\right)_{ij}\right| < \frac{\lambda_{2}}{\rho} \\ T^{k+1}_{ij} & \text{else } \end{cases}  $$

where $T^{k+1}_{ij}$ is defined as:

16 $$ {}T^{k+1}_{ij} = \left(\mathbf{Z}^{k+1} + \mathbf{U_{2}}^{k}\right)_{ij} - sgn\left(\left(\mathbf{Z}^{k+1} + \mathbf{U_{2}}^{k}\right)_{ij} \right) \frac{\lambda_{2}}{\rho}  $$

#### Solving for *Z*:

The optimization problem for **Z** defined in Eq. 6 can be equivalently written as:

17 $$ {}\begin{aligned} \mathbf{Z}^{k+1} &= \operatornamewithlimits{argmin}_{\mathbf{Z} \ge 0}\,\sum_{(u, v) \in E}\,-\log\left(1- \exp^{-C_{u}^{T}\mathbf{Z}C_{v}}\right)\\ &\quad+\,\sum_{(u, v) \notin E} C_{u}^{T}\mathbf{Z}C_{v} \\ &\quad+ \frac{p}{2}\left(\left\lVert\mathbf{Z} - \mathbf{Z_{1}}^{k} + \mathbf{U_{1}}^{k}\right\rVert^{2} + \left\lVert\mathbf{Z} - \mathbf{Z_{2}}^{k} + \mathbf{U_{2}}^{k}\right\rVert^{2}\right) \end{aligned}  $$

This problem can be efficiently solved via gradient descent using the backtracking line search for optimal step size selection which is based on satisfying the Armijo-Goldstein condition. Overall, our method is a combination of ADMM [34, 35] and gradient descent.

### Binning variant

When Hi-C data is binned, number of interactions between genomic regions *u* and *v* ($\phantom {\dot {i}\!}E^{'}_{uv}$) take integer values, as well as the number of marks at each region. In this case, resulting distribution $\phantom {\dot {i}\!}P(E^{'}_{uv} = k | \mathbf {X})$ becomes Poisson binomial distribution, and the probability of observing *k* Hi-C interactions out of $T = C_{u}^{T}\,\mathbb {1}\,C_{v}$ possible Hi-C interactions between bins *u* and *v* is:

18 $$ {}\begin{aligned} P(E^{'}_{uv} = k | \mathbf{X}) &= \sum_{F \in F_{k}}\,\prod_{(m, n, f_{mn}) \in F}\,(x_{mn})^{f_{mn}}\\ &\quad\prod_{(m, n, f^{c}_{mn}) \in F^{c}}\,(1-x_{mn})^{f^{c}_{mn}} \end{aligned}  $$

where summation is over all subsets of size *k*. *F*_*k*_ is set of subsets of size *k* ($F_{k} = \{(m, n, f_{mn})\,|\,(m, n) \in M^{2},\,f_{mn} \le c^{u}_{m}c^{v}_{n},\,\sum _{m, n \in M^{2}}\,f_{mn} = k\}$), and *F*^*c*^ ($F^{c} = \{(m, n, f^{c}_{mn})\,|\,(m, n) \in M^{2},\,\sum _{m, n \in M^{2}}\,f^{c}_{mn} = T - k\}$) is complement of *F*. *F*_*k*_ will contain $\frac {T!}{((T-k)!k!)}$ elements, the sum over which is infeasible to compute in practice unless *T* is small (e.g. if *T*=30, *F*_15_ contains over 10^20^ elements). However, there are other, more efficient ways to calculate $\phantom {\dot {i}\!}P(E^{'}_{uv} = k | \mathbf {X})$. By using Le Cam’s theorem [36], Eq. 18 can be approximated by Poisson distribution as in:

19 $$ P(E^{'}_{uv} = k | \mathbf{X}) = \frac{\lambda_{uv}^{k}e^{-\lambda_{uv}}}{k!}  $$

where $\lambda _{uv} = C_{u}^{T}\mathbf {X}C_{v}$ is a linear expression of **X**. This approximation is close to the exact formulation when *N*[*u*]*N*[*v*] is large and *x*_*mn*_*c*_*m*_*c*_*n*_ is small. When replaced *P*(*E*_*uv*_|**X**) in Eq. 1 with Eq. 19, resulting optimization problem is still nonconvex.

## Results

### Datasets and implementation

We utilize Hi-C data from human IMR90 cells [3] and human embryonic stem (ES) cells [14]. We also utilize the recent high-resolution Micro-C dataset from mouse ES cells [7]. Genome assemblies are obtained from the UCSC genome browser. We process species Hi-C sequencing reads by Juicer and as a result, retrieve the Hi-C interaction pairs from the genome assemblies. We obtain histone modifications and transcription factor binding sites for human and mouse from UCSC Encode [37] and NIH Roadmap Epigenomics [38]. We call chromatin mark peaks by MACS [39] utilizing previously reported parameters [40]. For unbinned scenario, we map each chromatin mark location to near Hi-C restriction sites. A chromatin mark belongs to a Hi-C restriction site if the mark is at most 100 away from the restriction site. In this paper, our analysis is for unbinned case unless mentioned otherwise. For the binned scenario, we bin transcription factor binding sites, ChIP-Seq histone modifications, Hi-C, and DNase-seq data at 1 kb resolution. Then, we estimate Reads Per Kilobase per Million (RPKM) in each bin by logarithmic transformation. This transformation decreases the high values distorting effects. When there are more than one replicates for a dataset, we minimize the effect of batch-related differences by averaging out the RPKM-level in each bin. Afterwards, we normalize such values into binary values by simple thresholding at 0.5.

We implement PROBC in Python, and solve several parts of optimization by L-BFGS method. Datasets and code are available at http://www.github.com/seferlab/probc. PROBC is reasonably fast: We can estimate interaction probabilities between marks in less than 15 minutes on human IMR90 cells even without binning. We follow fivefold nested cross-validation to prevent overfitting and optimize for regularization hyperparameters. When we train and test on different chromosomes, the outer step trains PROBC on all chromosomes except the chromosome to be predicted in fivefold nested cross-validation. Within each loop of outer step, we estimate the regularization hyperparameters by utilizing fourfold inner cross-validation. When we train and test on the same chromosome, we apply similar fivefold nested cross-validation on the single chromosome.

### CTCF and a small subset of histone modifications are critical for predicting hi-C interactions

**Fig. 1:**
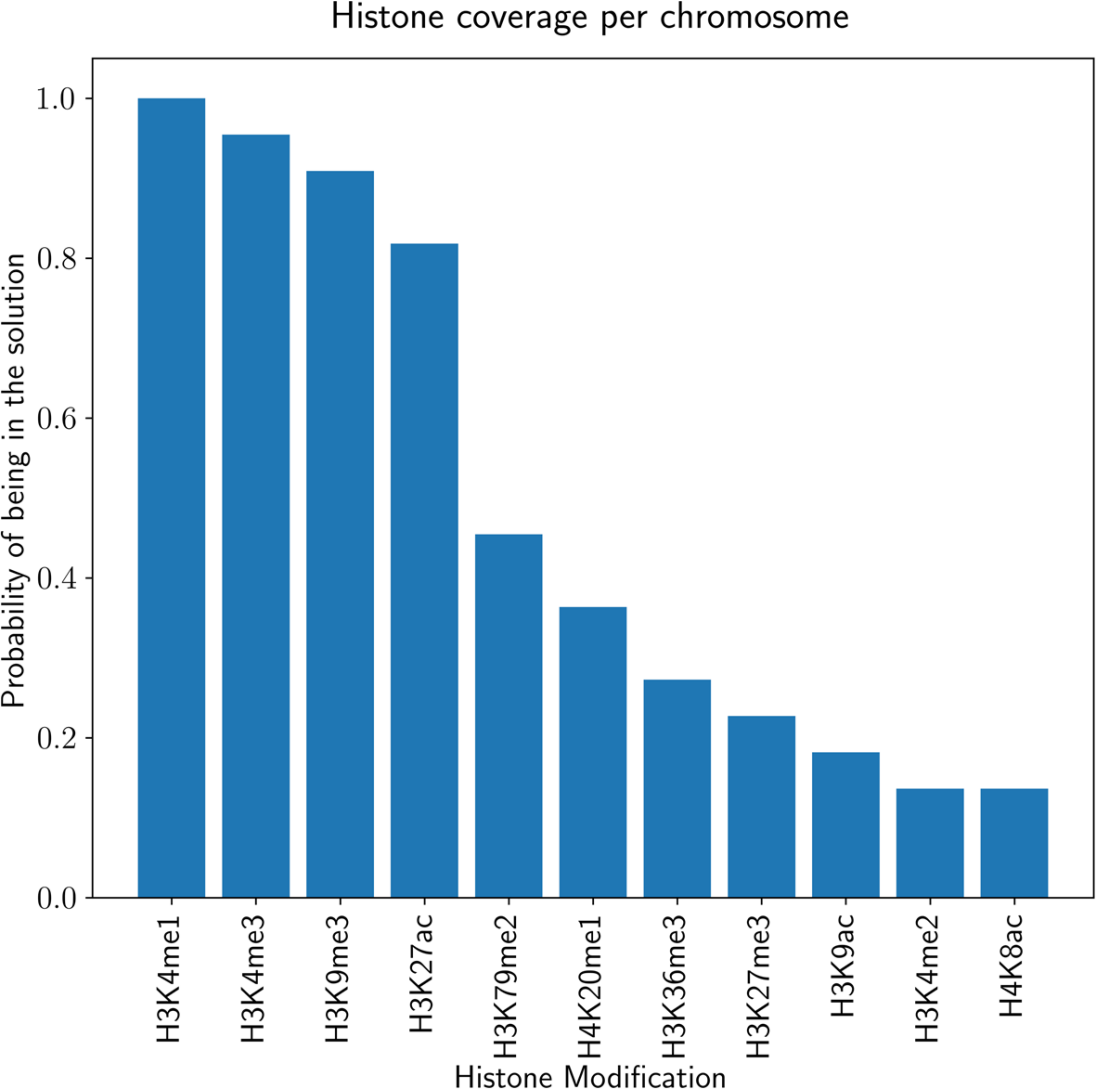
The probability of each histone modification showing up in PROBC solution where PROBC is applied to each chromosome independently on human ES cells

If we forbid the interactions between chromatin marks, we find that only 4 histone modifications (H3K27ac, H3K9me3, H3K4me3, H3K4me1) and CTCF out of total 32 chromatin marks (16 histone modifications, 16 TFBSs) are sufficient to explain the major proportion of Hi-C interactions in human ES cells. This is correct for both unbinned and 5 kb binned cases. These essential set of chromatin marks are significantly conserved when this procedure is repeated across mouse ES and human IMR90 cells with varying number of chromatin marks. Table 1 shows the histone modifications and transcription factor binding sites used in our experiments across species and cell types. Figure 1 displays the proportion histone marks end up showing up in the solutions per chromosome. When considered independently, H3K4me1, H3K4me3, H3K9me3, H3K27ac marks are also enough to explain most intra-chromosomal interactions.

**Table 1:** Histone modifications and transcription factor binding sites used in our experiments

Species & cell type	Histone modifications	Transcription factor binding sites
Human ES	H3K4me1, H3K4me3, H3K9me3, H3K27ac, H3K79me2, H3K36me3, H4K20me1, H3K27me3, H3K56ac, H3K23ac, H2AK5ac, H2A.Z, H3K9ac, H3K4me2, H4K8ac, H3K18ac	CTCF, SMC3, MAFK, CHD1, POLR2A, Dnase I, RAD21, CEBPB, MAZ, FOS, USF2, RCOR1, RFX5, ELK1, MXI1, NFE2L2
Human IMR90	H3K4me1, H3K4me3, H3K9me3, H3K27ac, H3K79me2, H3K36me3, H4K20me1, H3K27me3, H3K56ac, H3K23ac, H2AK5ac, H2A.Z, H3K9ac, H3K4me2, H4K8ac, H3K18ac	CTCF, SMC3, MAFK, CHD1, POLR2A, Dnase I, RAD21, CEBPB, MAZ, FOS, USF2, RCOR1, RFX5, ELK1, MXI1, NFE2L2
Mouse ES	H3K4me3, H3K27ac, H3K36me3, H3K4me1, H3K9me3, H3K27me3, H3K9ac	CTCF, POLR2A, EP300, MAFK, CHD2, HCFC1, ZC3H11A, ZNF384

Among the most important 5 chromatin marks, H3K4me1, H3K27ac are enhancer-specific marks. H3K9me3 is part of heterochromatin and is known to have repressive roles, whereas H3K4me3 is an activating mark. The same subset of histone modifications are important for human IMR90 cells as well, where activating mark H3K9ac and H3K36me3 that is associated with active gene bodies and elongation are also part of the solution. CTCF regulates the 3D structure of chromatin, and it is known to have roles in forming chromatin loops, TADs. It also defines the boundaries between active and heterochromatic DNA [41]. Among the rest of marks, H3K36me3 and H3K79me2 are known for their activator roles, whereas H3K27me3 is associated with polycomb repression similar to H3K9me3. For instance, Polycomb Repressive Complex PRC2 spreads the repression by binding to H3K27me3 [42]. PRC1 complex uses H3K27me3 to prevent the activation of RNAP II preinitiation complex. H3k27me3 is also statistically significantly depleted in TAD boundaries [11]. H3K36me3 is another mark of actively transcribed chromatin, which inhibitory effects depend on which subtype of PRC2 encounters the mark. Presence of PHF1 (characteristic of PRC2.1) is important for the spread of the H3K27me3 mark into H3K36me3-rich regions. Similarly, H4K20me1 is associated with transcriptional activation.

**Fig. 2:**
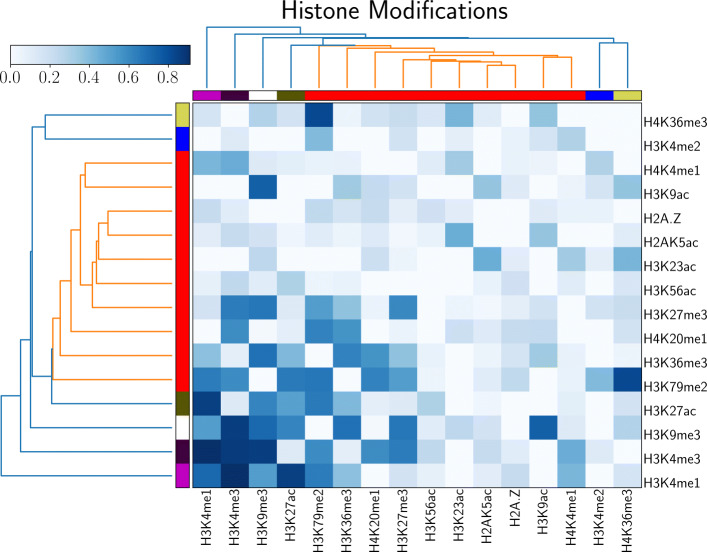
PROBC inferred genome-wide interaction probabilities between only histone modifications for human ES cells. *λ*_1_=2.5, and *λ*_2_=1.0 are found by fivefold nested cross-validation

Figure 2 show interaction probabilities between only histone modifications identified by PROBC, where *λ*_1_=2.5 and *λ*_2_=1.0 are found by fivefold nested cross-validation for genome-wide human ES cells. These parameters are important as otherwise PROBC assigns non-zero probabilities to all mark pairs. Previously identified 4 histone modifications have the highest interaction probabilities within themselves in explaining the given Hi-C interactions for human ES cells. Additionally, the interaction probabilities between H3K9ac-H3K4me2, H3K27me3-H3K27me3, H3K4me1-H3K4me1, and H3K9me3-H3K9ac modifications are also high. Both H3K9ac, H3K4me2 are known to exhibit activator features whereas H3K27me3 is known for its repressive characteristics. Results are similar for human IMR90 cells.

According to the dendrogram in Fig. 2, many modifications tend to be clustered together in terms of interaction profiles. For instance, repressive H3K9me3 and enhancer-specific modification H3K27ac tend to be similar to each other. Similarly, many modifications involving activating H3K4me2, H4K36me3 and Non-coding RNA (ncRNA)-repressive H2A.Z [43] also tend to be clustered suggesting the similar roles of different modifications on genome shape. The correlation between genome-wide interaction probability matrices of human ES and human IMR90 is significantly high 0.96. 5 histone interactions with the topmost probability difference between human ES and human IMR90 cells is shown in Table 2. Among these interactions with the largest probability difference, H3K27me3 - H3K36me3, H3K9me3 - H3K79me2, and H3K9me3 - H4K20me1 are between repressive histone modifications and modifications associated with active gene bodies and elongation. The remaining two modifications are between repressive and activating modifications. These results suggest the interplay between different types of modifications can contribute to different three-dimensional genome shapes among different cell types of a given species. Overall, these probabilities can also be interpreted as latent biases in Hi-C, and can be used to filter out the noise in Hi-C.

**Table 2:** 5 histone interactions with the topmost interaction probability difference between human ES and human IMR90 cells

Interactions	Human ES	Human IMR90	Abs. diff.
H3K27me3 - H3K36me3	0.196	0.25	0.05
H3K9me3 - H3K79me2	0	0.04	0.04
H3K9me3 - H3K9me3	0.71	0.67	0.04
H3K9me3 - H4K20me1	0.12	0.09	0.03
H3K4me2 - H3K4me3	0.06	0.04	0.02

### PROBC finds a small subset of transcription factor binding sites predictive of hi-C interactions

**Fig. 3:**
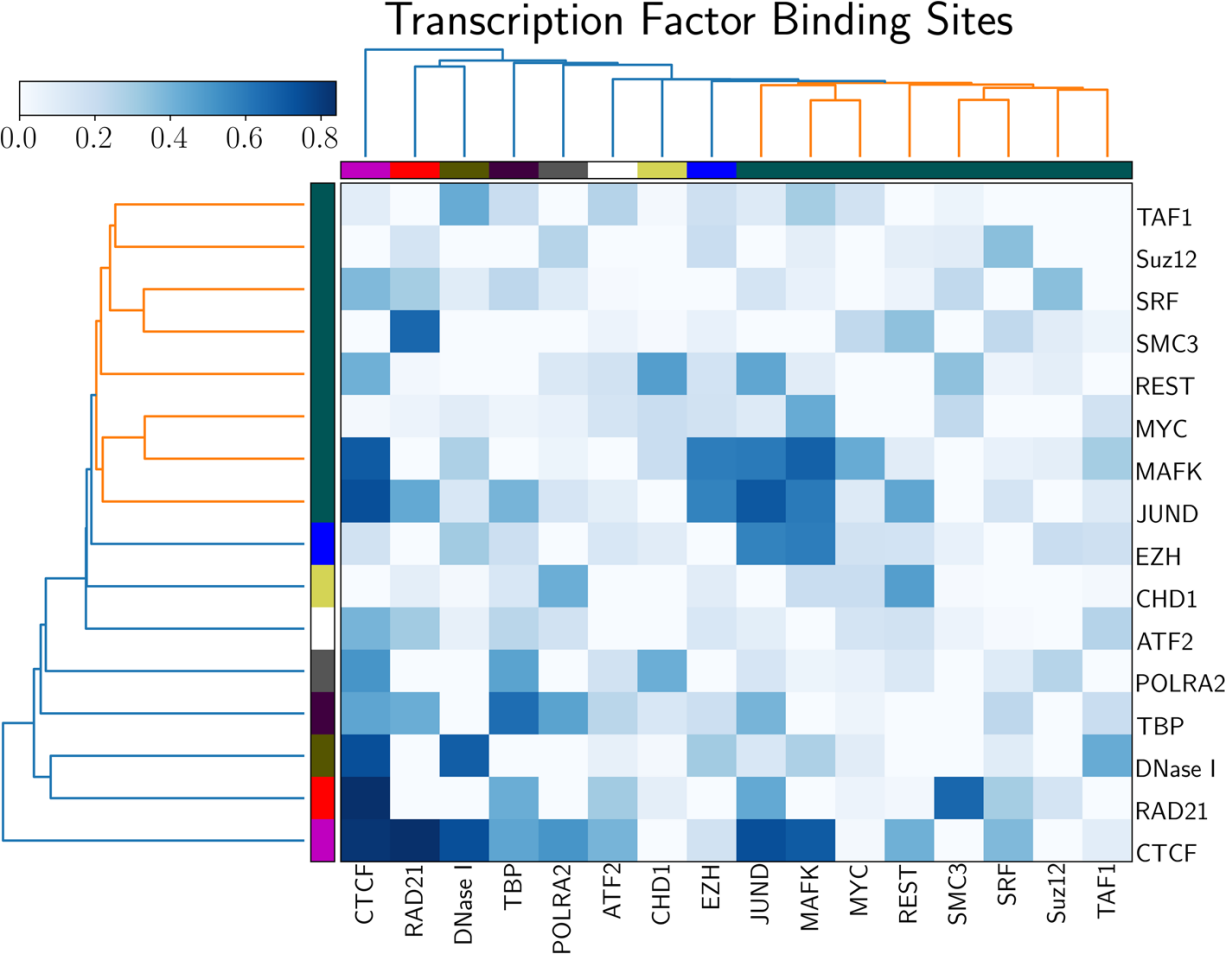
PROBC inferred genome-wide interaction probabilities considering only transcription factor binding sites for Human ES cells

We also examine the independent effect of transcription factor binding sites in human ES cells. PROBC reveals CTCF, RAD21, DNase I, TBP, POLRA2, Suz12, EZH2 in explaining the given Hi-C graph which genome-wide probabilities are shown in Fig. 3. In this case, the initial part of the probability matrix is not as probable as in Fig. 2. The accuracy of transcription factor binding sites is lower than the one for epigenetic modifications. For instance, almost 12 transcription factor binding sites give the same Hi-C coverage performance as 4 histone modifications. Among the identified transcription factor binding sites, RAD21 encodes an evolutionary conserved DNA double-strand break repair protein, which is a structural component of the highly conserved cohesin complex [44]. DNase I hypersensitive sites identify genome regions with active genes [45], which is due to them being characterized by accessible, open chromatin. TBP binds to DNA during the transcription preinitiation complex formation [46]. When histone modifications H3K27me3 and H3K9me3 are not considered, Suz12 and EZH2 components of Polycomb Repressive Complex PRC2 start to appear in both solutions. Suz12 and EZH2 are responsible for the methylation activity of PRC2, which can bind to H3K27me3 and repress neighboring nucleosomes. The other complex PRC1 uses H3K27me3 to inhibit the activation of RNAP II preinitiation complex [47]. Lastly, POLRA2 gene encodes the largest subunit of RNA Polymerase II, which is responsible for synthesizing mRNA in eukaryotes.

Among interactions between transcription factor binding sites, RAD21 and SMC3 interaction is highly probable. Both proteins are part of Cohesin complex, and SMC3 is present in all cohesin complex whereas there are multiple paralogs for RAD21. RAD21 is an evolutionarily conserved in all eukaryotes from budding yeast to human. This interaction is also highly probable in human IMR90 cells. Similarly, high probability interaction between TBP binding sites also suggests the importance of binding to TATA box in genome shape formation. Interestingly, CTCF-JUND and CTCF-MAFK interactions are also highly probable which do not belong to the same protein complex. Roles of both MAFK and JUND are different than CTCF: MAFK is basic region and leucine zipper (bZIP)-type transcription factor, whereas JUND protein is a member of the JUN family, and a functional component of the AP1 transcription factor complex. These interactions need to be carefully examined to further develop novel insights about genome shape formation.

### PROBC predicts Hi-C interactions and genomic structures from chromatin marks

**Fig. 4:**
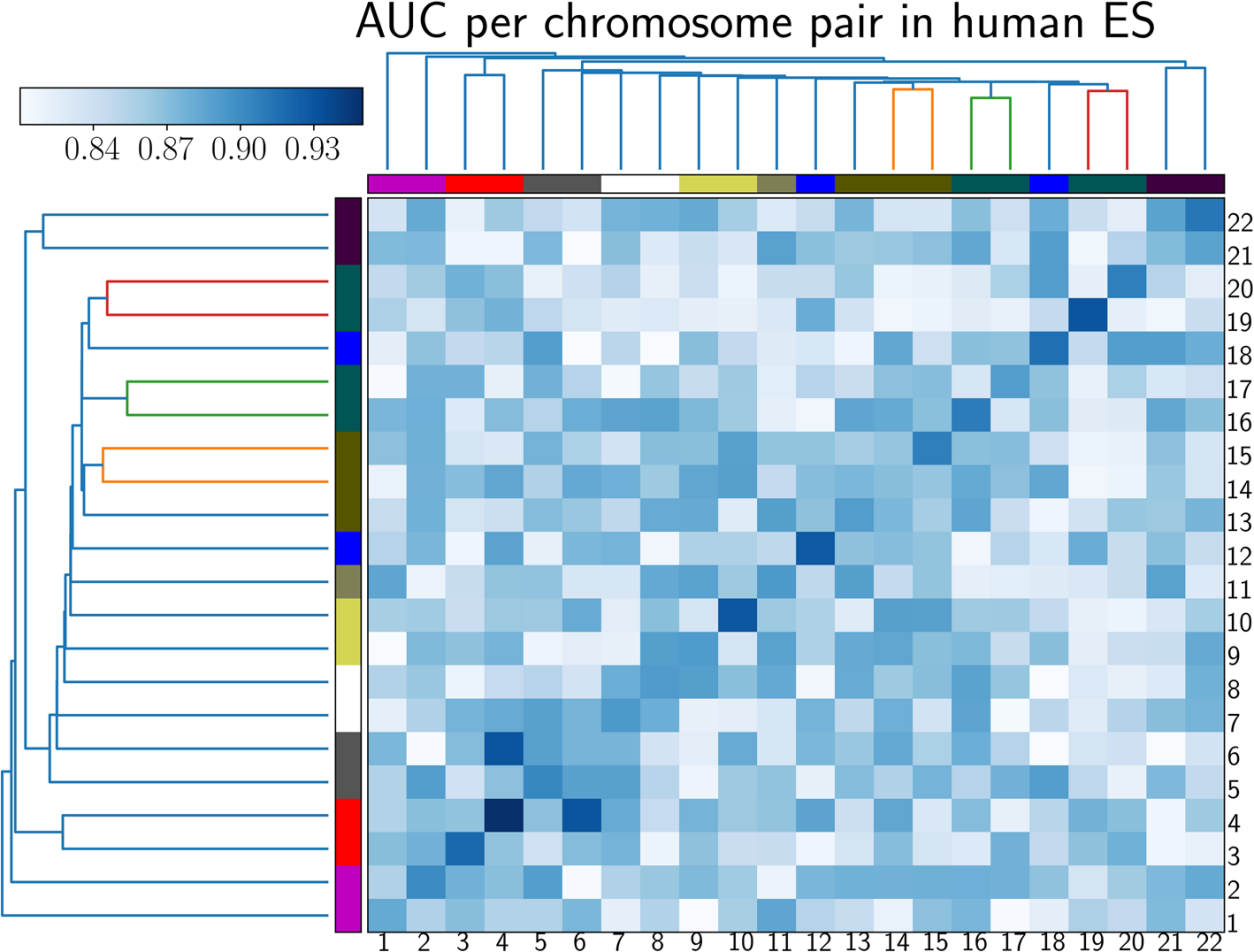
AUC scores in predicting Hi-C interactions for each pairs of chromosome on human ES cells. We apply fivefold cross-validation to each pairs of chromosome

**Fig. 5:**
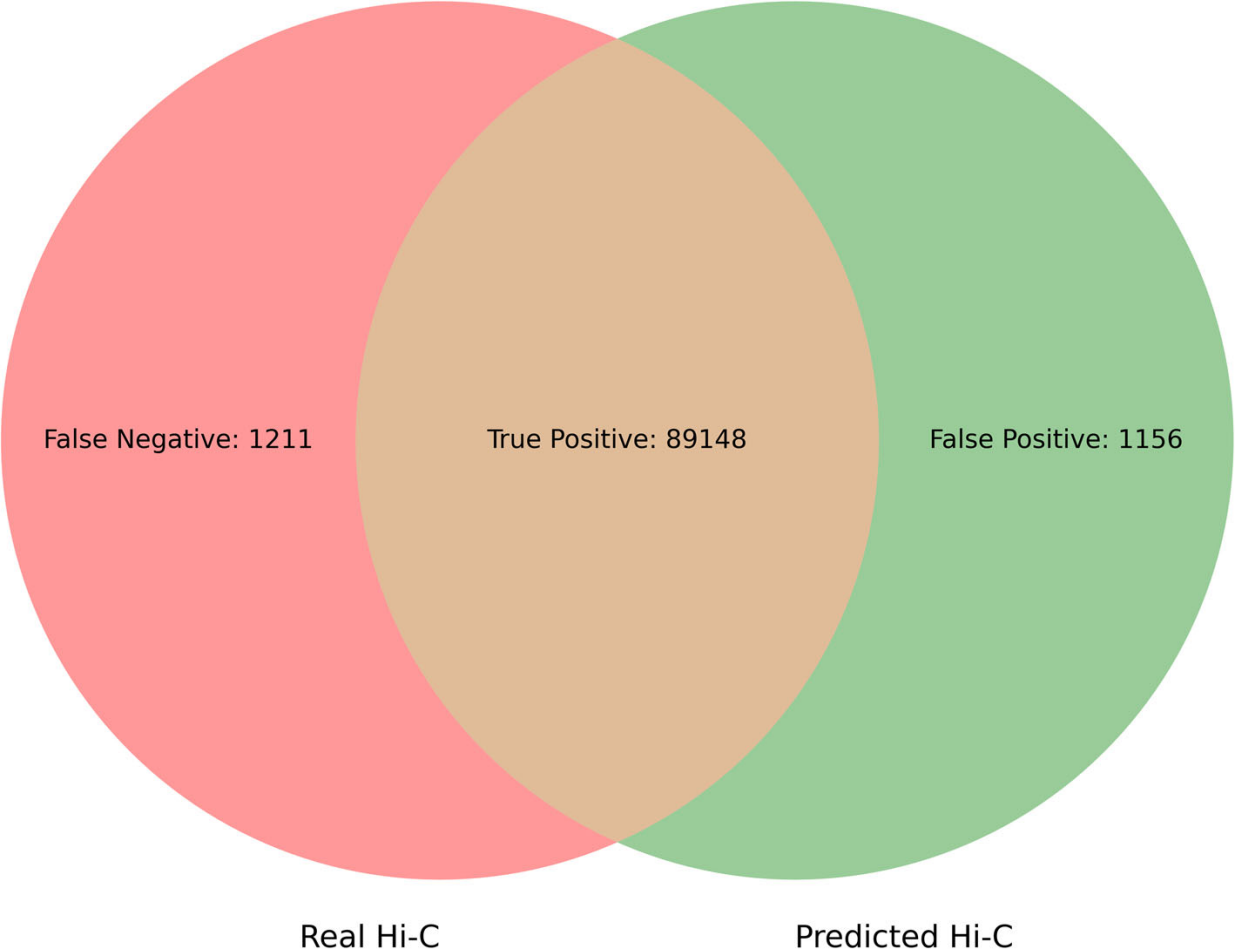
Real vs Predicted Hi-C Interactions in human ES cells chromosome 4

**Fig. 6:**
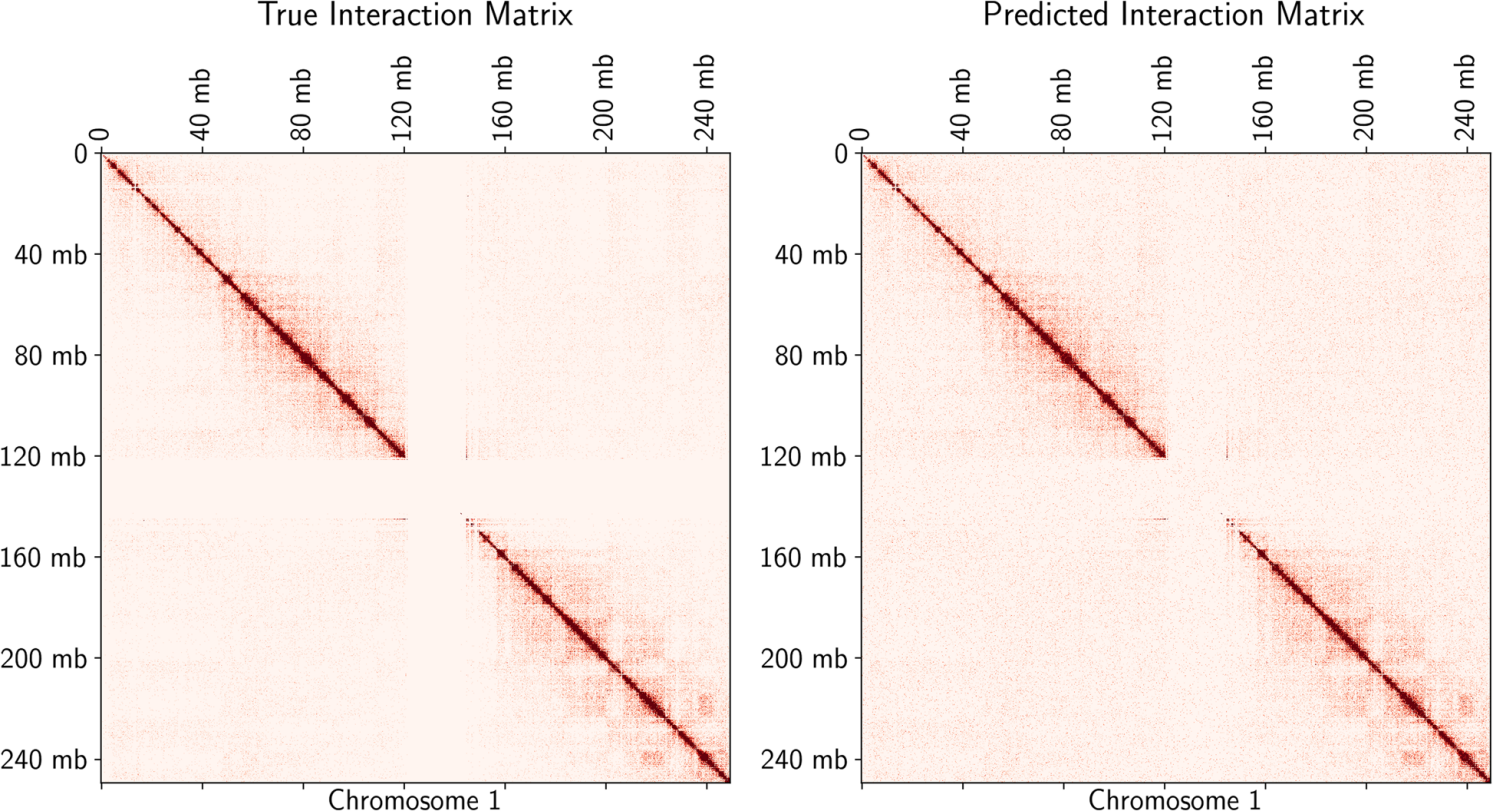
Comparison of predicted vs real Hi-C interaction matrices at 10 kb resolution for human ES cells chromosome 1

On human ES cells, PROBC can detect false positive interactions and predict novel Hi-C interactions. In such experiments, fivefold nested cross validation is applied independently to each chromosome. We first predict Hi-C interactions from previously identified H3K27ac, H3K9me3, H3K4me3, H3K4me1, and CTCF. Then, we obtain ROC curves by decreasing the Hi-C interaction probability threshold from 1 to 0. According to the pairwise comparison matrix in Fig. 4, 4th chromosome shows the best performance with 0.95 ROC AUC where AUC (Area Under Curve) score shows the tradeoff between false positive and false negative interactions. Figure 5 shows the details of such prediction by Venn Diagram for chromosome 4. Similarly, we also visually compare the predicted interaction matrix with the true interaction matrix of human ES cells chromosome 1 at 10 kb resolution as seen in Fig. 6. According to cross-chromosomal experiments, PROBC performance declines but it is still reasonable when we train it on one chromosome and test on a different chromosome. As an example, we obtain ROC AUC score of 0.86 when we train with Hi-C interactions on the 6’th chromosome and predict interactions on the 4’th chromosome. The similarity of the chromatin marks found as important across chromosomes suggests the similarity of main properties controlling chromosomal contact formation. Although, there can still be close-grained differences which have not been caught by PROBC.

**Fig. 7:**
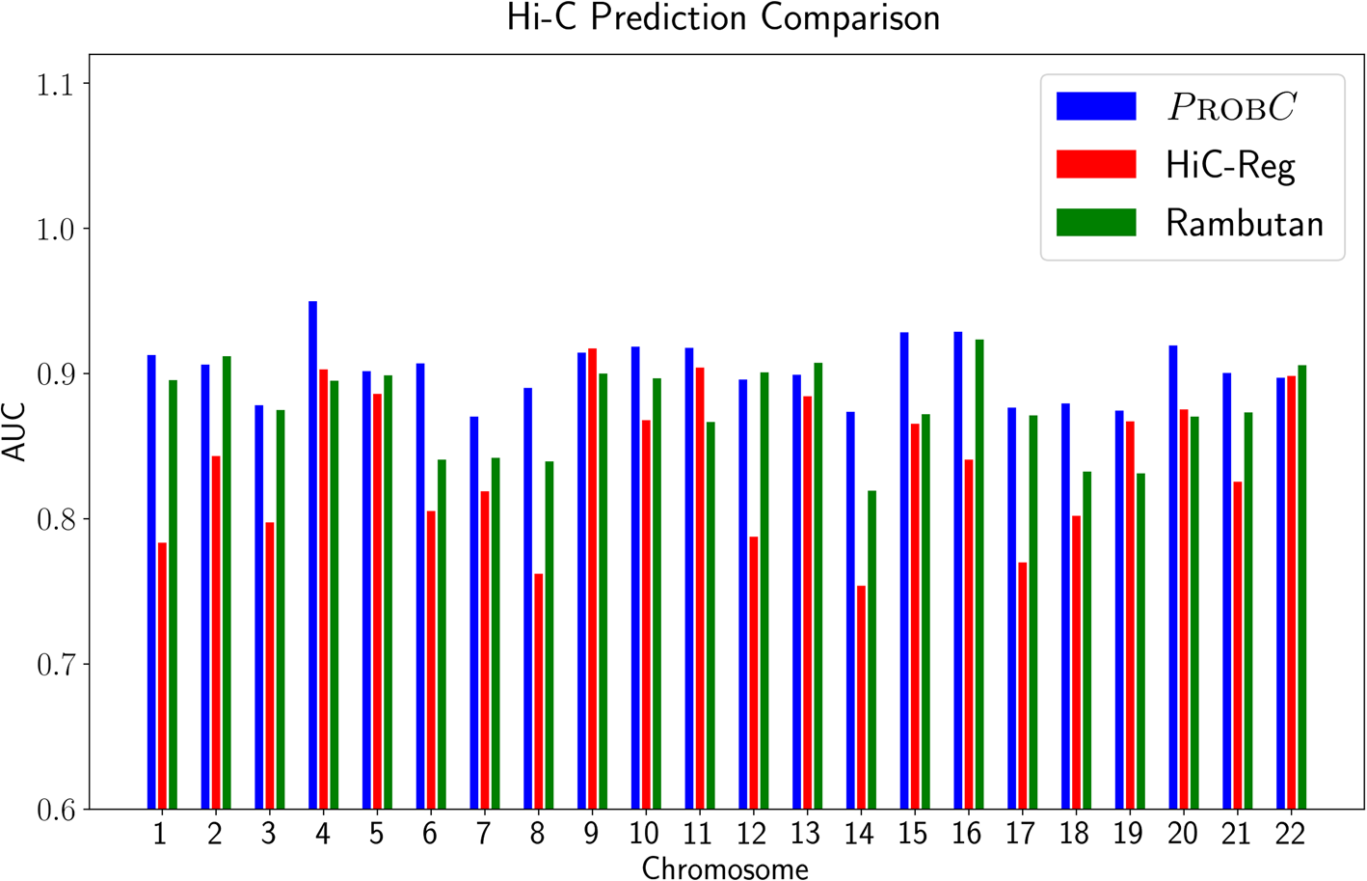
Hi-C prediction performance comparison of PROBC, HiC-Reg, and Rambutan by AUC score on human ES cells

PROBC outperforms random-forest based approach HiC-Reg, and convolutional neural network-based approach Rambutan for most of the intra-chromosomal interaction prediction in terms of ROC AUC score on human ES cells as in Fig. 7. We cannot compare PROBC with EpiTensor as EpiTensor constructs interactions jointly across multiple species from chromatin datasets, whereas the other methods are based on individual species. While comparing with HiC-Reg in terms of ROC AUC, we divide each count value by the sum of the counts to normalize the HiC-Reg output. The performance difference is the most apparent in chromosome 4. The outperformance of PROBC still exists for human IMR90 cells. Additionally, both Spearman and Stratum-adjusted correlation coefficient [48] between PROBC and HiC-Reg, EpiTensor, Rambutan performances is moderate, showing the impact of the difference between the nonlinear approaches these methods are based on. Variables identified as important by HiC-Reg and PROBC overlap. For instance, H3K27ac, H3K9me3, and H3K4me3 also appear to be important according to out of bag (OOB) variable importance measure, that measures the change in OOB error by permuting the feature values. Rambutan performs slightly better than HiC-Reg as it additionally uses nucleotide sequence data. However, in terms of other datasets, it is limited to Dnase I signal.

**Fig. 8:**
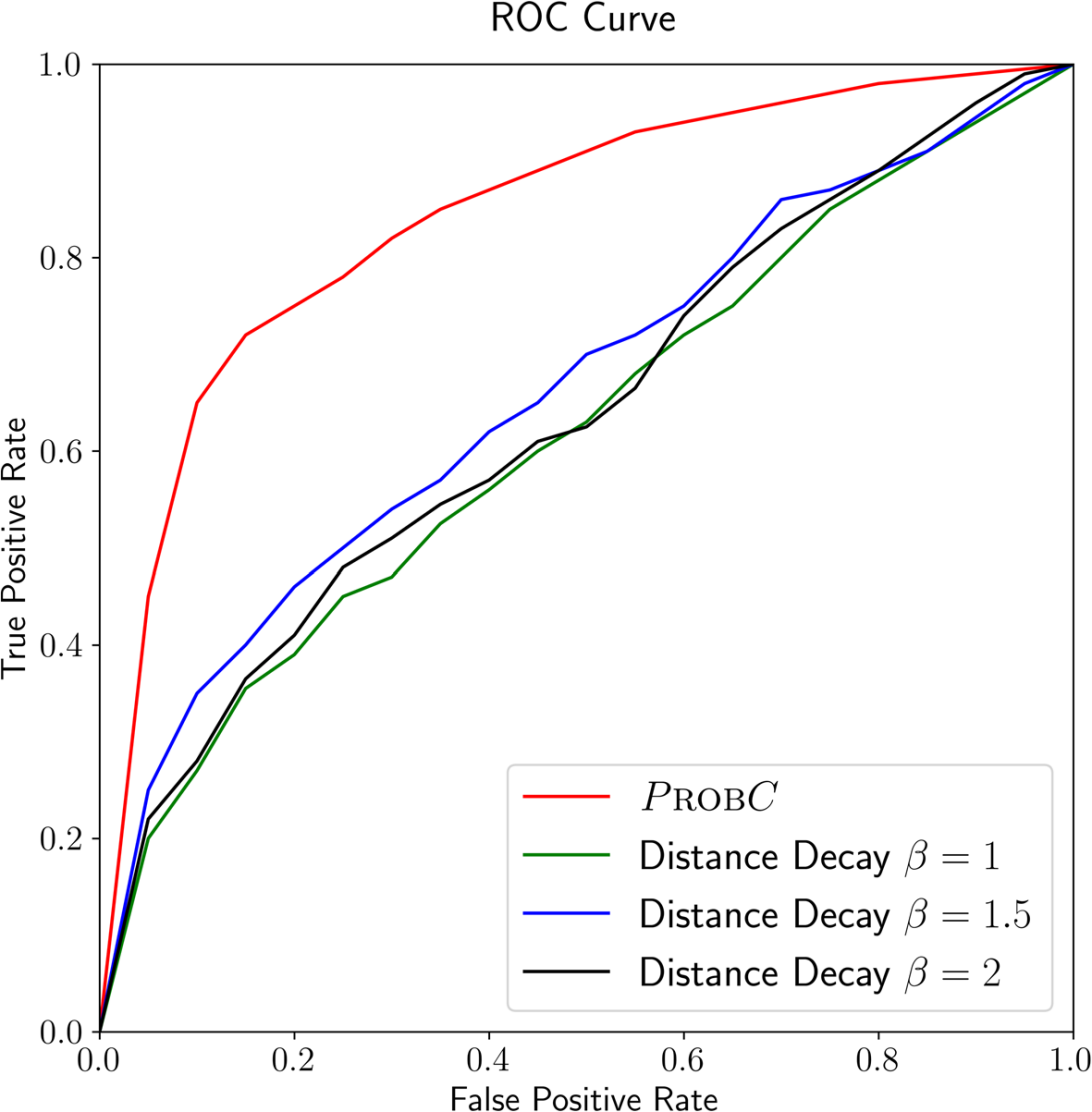
ROC Curve for Hi-C prediction performance comparison of PROBC and distance decay heuristics with various exponents on human ES cells chromosome 1

We also compare PROBC predictions with multiple distance decay-based prediction baselines as seen in Fig. 8 for chromosome 1 in human ES cells in terms of ROC curve, where the probability of interaction between two segments *u* and *v* is inversely related to their genomic distance. In more detail, *P*(u and v interact with each other)=*α* *d*(*u*,*v*)^*β*^ where *d*(*u*,*v*) is the genomic distance between segments *u* and *v*, and optimal *α* is learned over the training set. in our case, we test the performance for various *β* exponent values for variety of thresholds as in the figure. According to the figure, PROBC clearly outperforms all three distance decay baselines with *β*=1,1.5,2. Among the baselines, distance decay baseline with *β*=1.5 performs the best, but its AUC score is still remarkably lower than PROBC.

**Fig. 9:**
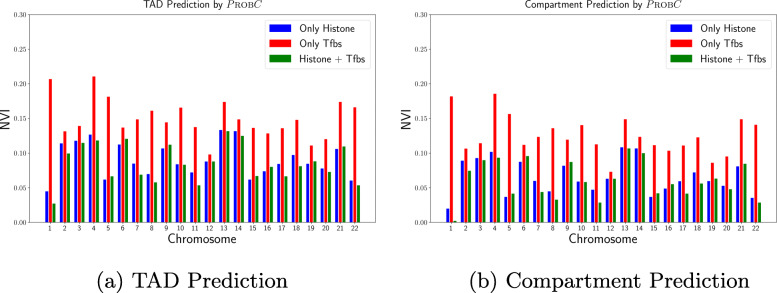
TAD and Compartment prediction performance of PROBC by Normalized Variation of Information across only histone modifications, only transcription factor binding sites, and for combination of both on human ES cells

In addition to Hi-C prediction, Fig. 9a-b shows PROBC’s performance for TAD and compartment domains prediction on human ES cells respectively. The performance is evaluated for only histone modifications, only transcription factor binding sites, and for combination of histone modifications and transcription factor binding sites. We compare the TAD and compartment domains prediction across different chromatin mark combinations by Normalized Variation of Information (NVI) [49], which quantifies the distance between predicted and true TAD/compartment domain partitions with higher score denotes a worse performance. Domain prediction software Armatus [10] is used to identify TADs over predicted interactions. Similarly, the compartment identification method in [50] has been used to identify A/B compartment domains over predicted interactions in 1 kb resolution. This compartment detection method retrieves A/B compartments from the signs of the first eigenvector of the interaction matrix, without requiring any additional parameters. The compartment prediction results are relatively robust across different resolutions; When we use both transcription factor binding sites and histone modifications, NVI scores are close to 0.05 for matrices binned at 10 kb and 40 kb resolutions. PROBC trained only with histone modifications outperforms the case it was trained only with transcription factor binding sites for both TAD and compartment domain prediction. However, the best TAD prediction performance is observed when we combine both mark datasets.

We have also analyzed the breakdown of TAD prediction in terms of TAD boundaries as shown in Table 3 across a number of resolutions in all chromosomes of human ES cells. Even at a higher resolution as 1 kb, PROBC followed by TAD prediction method Armatus can correctly predict 12.31*%* of TAD boundaries correctly. If we look into the false positive boundaries, we found most of them to be near the true TAD boundaries, which can also be inferred from lower NVI scores. The accurracy of TAD prediction increases if we make the matrices smaller by decreasing the resolution. For instance, we can predict 69.83*%* of TAD boundaries correctly at 100 kb resolution.

**Table 3:** Real vs Predicted TAD boundaries in human ES cells across all chromosomes

	Resolution			
Metrics	1 kb	5 kb	40 kb	100 kb
True Positives	10,245	13,452	12,546	4,724
True Positive Rate	12.31*%*	27.59*%*	62.31*%*	69.83*%*
False Negatives	72,779	35,304	7588	2,042
False Negative Rate	87.69*%*	72.41*%*	37.69*%*	30.17*%*
False Positives	41,123	21,134	6,020	672
Predicted TAD Boundaries	51,368	34,586	18,566	5,396
True TAD Boundaries	83,204	48,756	20,134	6,766

### PROBC can predict perturbations in 3D architecture

As PROBC indirectly models the chromatin marks contribution to three-dimensional shape via chromatin interactions, we have investigated whether PROBC can forecast modifications to 3D structure explained via perturbations over chromatin marks. We have especially focused on samples where significant chromatin marks are removed due to structural variations. Despang et al. [51] has investigated the fusion of TADs when CTCF binding sites are deleted in vivo at the Sox9-Kcnj2 locus of the mouse embryonic limb. They have utilized the promoter capture Hi-C data in the E12.5 mouse limb to display the changes in structure after removing principal CTCF binding sites (mm9, GSE78109, GSE125294). In the unmodified cells, Sox9 and Kcnj2 are part of different TADs. Once successive 4 CTCF sites are deleted inside a 15 kb boundary region, the existing TAD boundaries do not appear any more and TADs are combined together. TADs are fused more thoroughly once the whole set of CTCF binding sites between Sox9 and Kcnj2 are removed. Overall, according to those experiments, TAD fusion is disclosed as a result of removal of crucial CTCF binding sites inside TADs as well as across their boundaries.

We have analyzed whether those modifications in the TAD structure can be predicted by PROBC once we have perturbed the input CTCF dataset. We trained PROBC by using human ES cells dataset, and we made predictions across species in mouse ES cells. PROBC has normally predicted a robust TAD boundary between Sox9 and Kcnj2, putting these into separate TADs. Once we mask the four CTCF binding sites at the boundary and additionally mask the whole set of CFCF binding sites, results predicted by PROBC are consistent with the true experiments in which 2 separate TADs are slowly combined into a single TAD. Furthermore, we have investigated the association between CTCF binding sites and formation of TADs via feature attribution approaches. We have estimated SHAP values [52], and averaged them out for bins. We have found that CTCF binding sites at TAD boundary are important for TAD segregation across the unperturbed experiments. On the other hand, by using the perturbed CTCF dataset, the attribution scores have focused more on distant parts at fused TAD boundaries.

In another experiment, we have investigated whether changes in the structure due to genomic deletions can be predicted by PROBC. Yang et al. [53] have found that FLT3 gene is upregulated in acute lymphoblastic leukemia (ALL) cell datasets with a 13q12.2 deletion. They have also associated that expression increase to enhancer hijacking and chromatin’s organization. They have found that regulation of FLT3 is achieved via 3 functional regulatory elements over 13q12.2 segment: DS1 (chr 13:28,100,363−28,100,863), FLT3’s promoter; DS2 (chr 13:28,135,863−28,140,863); and DS3 (chr 13:28,268,863−28,269,363) which belongs to PAN3 gene’s intron. Across non-cancer hematopoietic cells, the interaction between DS2 and DS1 elements mainly control FLT3 gene for partition into 2 TADs, where DS3 is positioned over a closer TAD and DS2 element intersects with the TAD boundary. When DS2 has been dropped due to delection of 13q12.2 region, Yang et al. [53] has noticed that longer-range interaction between DS3 and DS1 becomes more stronger, and two closer TADs are fused.

In our evaluatuon, simulation of this deletion is achieved by cutting out DS2 region from all chromatin marks input datasets and predicting the interaction matrix via PROBC. In this case, all chromatin marks corresponding to the deleted region were removed, and the remaining two partitions were added together. TADs inferred by PROBC are compatible with the true discoveries. Without 13q12.2 deletion, a smaller TAD segregation between PAN3 and FLT3 genes are predicted by PROBC, in line with the ground truth. However, once 13q12.2 is deleted, PROBC finds that 2 TADs are fused together and number of interactions between PAN3 and FLT3 increases.

### PROBC can accurately predict cross-cell types and cross-species interactions

**Fig. 10:**
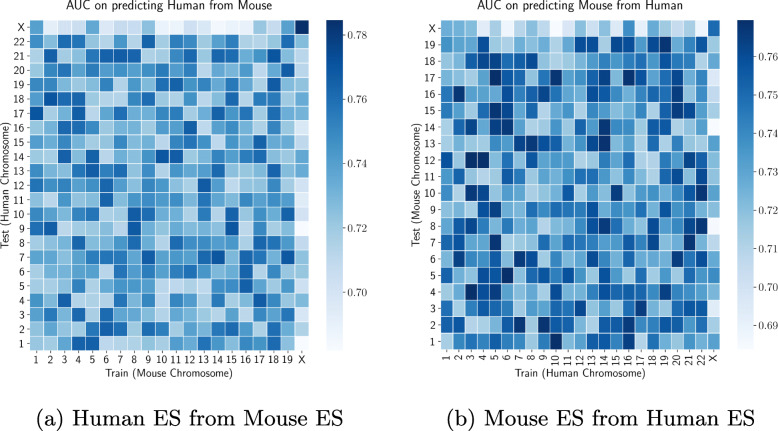
AUC score for interaction prediction a) human ES from mouse ES, b) mouse ES from human ES cells. We apply fivefold cross-validation to each pairs of chromosome

**Fig. 11:**
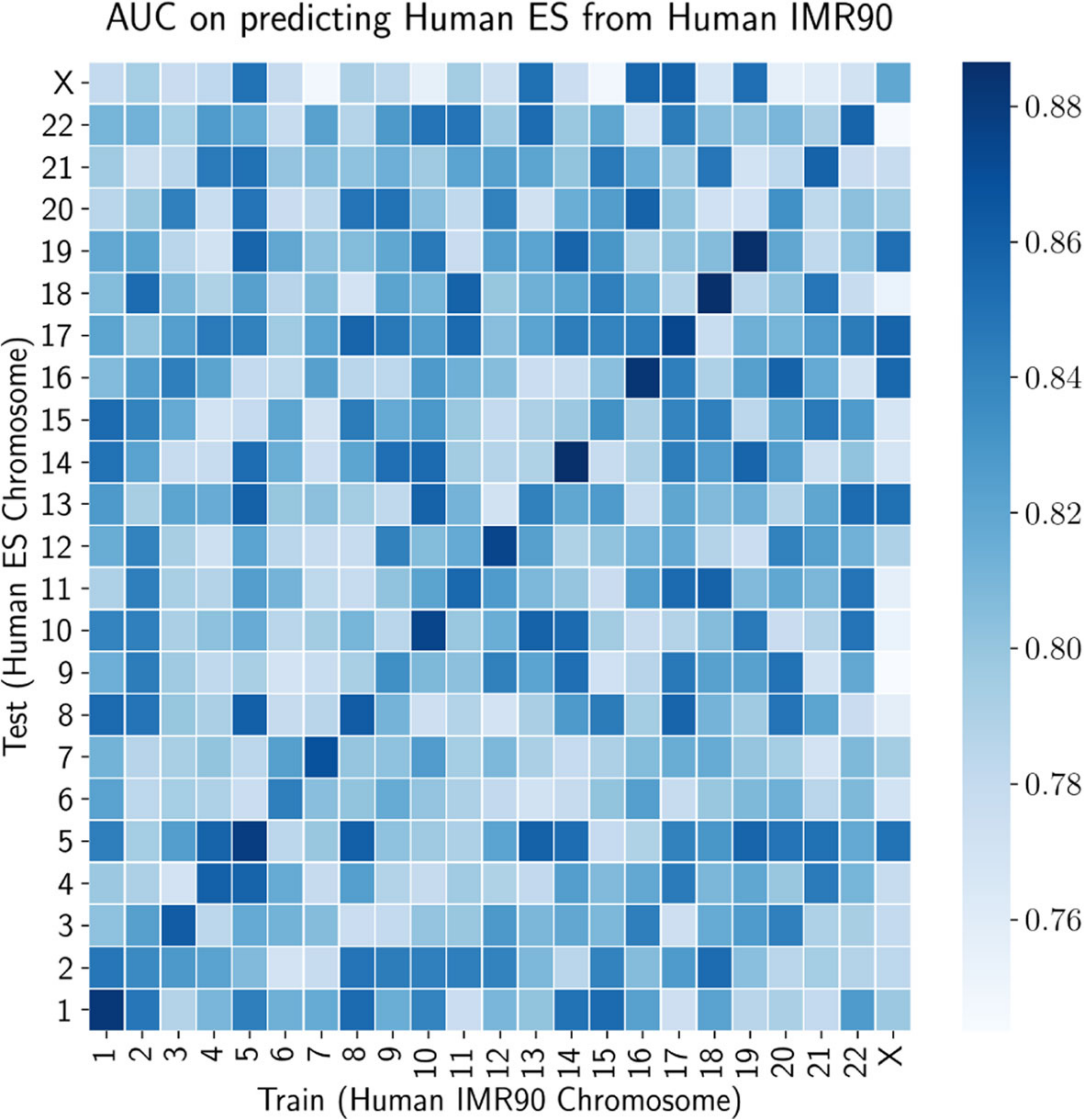
AUC score for interaction prediction on human ES from human IMR90. We apply fivefold cross-validation to each pairs of chromosome

We predict Hi-C/Micro-C interactions on mouse ES and human ES chromosomes over PROBC trained with chromosome-wide human ES and mouse ES cells respectively. We apply fivefold cross validation to Hi-C, histone modifications and transcription factor binding sites datasets. Figure 10a displays AUC score across 23 human chromosomes (22 + X chromosomes). The worst performance is observed for X chromosome, and PROBC can predict inter-chromosomal interactions less accurately than the intra-chromosomal interactions. Similar prediction analysis on mouse ES cells while training PROBC over human ES chromosomes is shown in Fig. 10b. Additionally, we investigate the impact of cell types in Hi-C interaction prediction as in Fig. 11. The prediction performance between human IMR90 and human ES is better than the prediction between species suggesting that common subset of chromatin marks explain genome shapes of different cell types in the same species. There is no significant performance difference between training on human ES vs. IMR90.

Prediction performance between species is lower than the performance between cell types on the same species, showing effective histone modifications may slightly differ across species. Results show the importance of species-specific interactions between histone modifications in genome shape, since the performance decreases even when modifications are transferred from a closer species.

### PROBC is robust to changes in *λ*_1_ and *λ*_2_

**Fig. 12:**
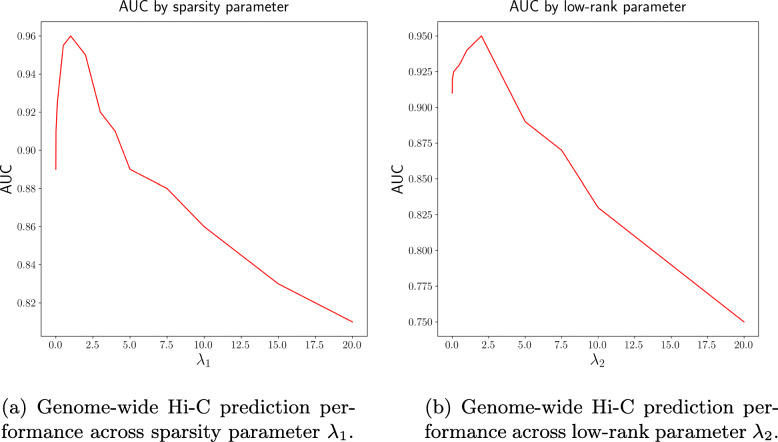
PROBC is robust to changes in *λ*_1_ and *λ*_2_

PROBC is robust to changes in sparsity parameter *λ*_1_ and low-rank parameter *λ*_2_ as seen in Fig. 12a-b respectively. We obtain AUC score by decreasing the Hi-C interaction probability threshold from 1 to 0. A reasonable increase of *λ*_1_ does not significantly decrease the quality of the predictions, while it leads Hi-C data to be explained by fewer chromatin marks. A subset of previously identified histone modifications (H3K27ac, H3K9me3, H3K4me3, H3K4me1) and CTCF appear in the solution when *λ*_1_≥5. On the other hand, increasing the low-rank parameter *λ*_2_ ensures block diagonal/communities structure of the mark interactions matrix, but decreases genome-wide performance especially when *λ*_2_≥10. Robustness of PROBC across parameters shows that the reported performance and generated insights in previous sections are quite reliable.

### PROBC is stable to enzyme replicates, robust across resolution parameters

**Fig. 13:**
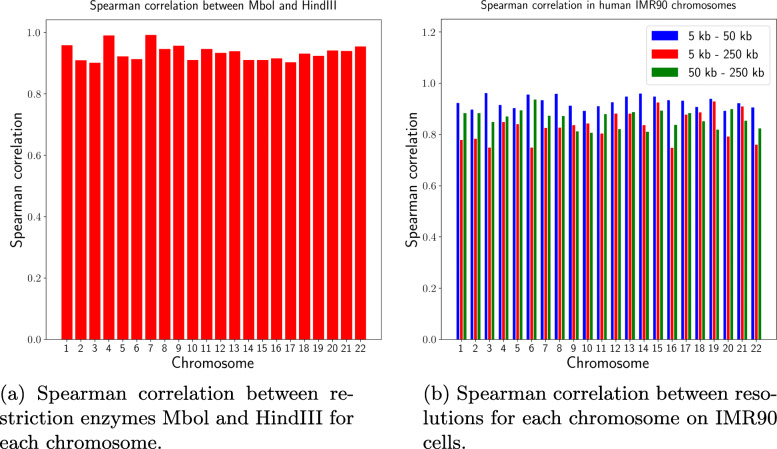
PROBC is stable to enzyme replicates, robust across resolution parameters

Hi-C experiments utilizes a restriction enzyme such as Mbol and HindIII to cleave the DNA after cross-linking, and different restriction enzymes can be used to analyze the sequence dataset multiple times for this purpose. Even though the resulting restriction fragments differ, the complete genome architecture and thus epigenetic and transcription marks should be the same from such replicate experiments. Therefore, we assess the stability of PROBC regarding the similarity of the interaction probabilities inferred from two replicate Hi-C experiments that differ only in the choice of restriction enzyme. Specially, we apply PROBC to two enzyme replicates, Mbol and HindIII, carried out in human IMR90 cells. We evaluate the stability by Spearman correlation between the two pairwise interaction probabilities matrices of the pairs of histone modifications on each chromosome as in Fig. 13a. Overall, correlation is broadly the same across chromosomes, and the correlation between different restriction enzymes on chromosomes 4 and 7 is slightly higher than the rest.

Similarly, our methods are stable to changes in resolution as seen in Fig. 13b, where Spearman correlation between binned pairs of interaction probabilities at 5 kb, 50 kb, 250 kb resolutions are calculated on IMR90 cells for each chromosome. Interaction matrices between chromatin marks are most similar between 5 kb and 50 kb resolutions, where the matrices tend to be different for lower resolutions (250 kb). For lower resolution, due to the binning effect, we tend to see almost all histone modifications in every bin so the expressive power of our methods decreases significantly.

## Conclusion

We explore the 3D genome organization by investigating how individual chromatin marks such as histone modifications and transcription factor binding sites as well as interactions between these chromatin marks explain Hi-C interactions. We come up with a novel probabilistic generative model-based method PROBC to optimally decompose Hi-C interactions in terms of these chromatin marks at genome and chromosome levels. Via our method, we find a subset of histone modifications and transcription factor binding sites to be predictive of Hi-C interactions and TADs across human, mouse, and different cell types. Given Hi-C data is still limited to certain species, accurate prediction of Hi-C interactions at a high resolution without integrating Hi-C data is mainly useful to analyze the 3D genome shape on such species. The similarity of the chromatin marks found as important across chromosomes suggests the similarity of main properties controlling chromosomal contacts. In summary, the analysis performed and all predictions made by PROBC in this work give good insights in exploring the 3D chromatin organization. We find that identified chromatin marks alone carry almost enough information to predict chromosomal structures.

In the future, we can extend the proposed method to recent multilocus chromatin interaction datasets by using a hypergraph instead of a graph. The assumption of same interaction probability in PROBC between marks might be relaxed as it is still open whether chromatin marks work differently depending on genome region. Lastly, we can extend PROBC to directly carry out a differential analysis on multiple tissues to predict differential interactions.

## Data Availability

The source code and data are available at http://www.github.com/seferlab/probc.
