# Correction: ProbC: joint modeling of epigenome and transcriptome effects in 3D genome

**DOI:** 10.1186/s12864-024-10947-2

**Published:** 2024-11-18

**Authors:** Emre Sefer

**Affiliations:** Department of Computer Science, Ozyegin University, Istanbul, Turkey

**Correction: BMC Genomics 23, 287 (2022)**

10.1186/s12864-022-08498-5

Following publication of the original article it was reported there was a missing declaration of funding in the Funding declaration.

The updated Funding declaration with the newly added text in bold is given below.

**Funding**

Ozyegin University Research Grant. **This research was funded by TUBITAK (Scientific and Technological Research Council of Turkey) 3501 Project with grant number 122E706**.

The original article has been updated.

